# Supplementary material for: Control of transmembrane charge transfer in cytochrome c oxidase by the membrane potential
Source: Nat Commun. 2018 Aug 9;9:3187. doi: 10.1038/s41467-018-05615-5 (PMC6085328; doi:10.1038/s41467-018-05615-5)
Supplement: Supplementary file 1 — Supplementary Information [file 41467_2018_5615_MOESM1_ESM.pdf]

Supplementary material

Control of transmembrane charge transfer in cytochrome *c* oxidase by the membrane potential

Markus L. Björck and Peter Brzezinski*

Department of Biochemistry and Biophysics, The Arrhenius Laboratories for Natural Sciences, Stockholm University, SE-106 91 Stockholm, Sweden.

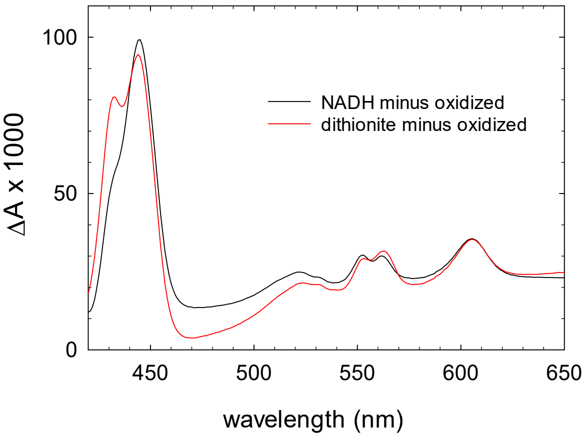

Supplementary Figure 1. Orientation of the respiratory chain the SMPs. The SMPs were reduced with NADH (black difference spectrum) and then by dithionite (red difference spectrum) under anaerobic conditions. The spectrum of the oxidized sample was subtracted from that obtained after each addition. The redox difference spectrum after addition of NADH reflects the SMP population in which the NADH-binding site is oriented to the outside. Dithionite was added until no further absorbance changes were observed, i.e. the entire SMP population was reduced. The larger peak at 430 nm and 560 nm is due to reduction of the low-potential heme b_L with dithionite (this is an effect of midpoint potential rather than orientation). Experimental conditions: 0.9 mg/ml SMPs, 5 mM NADH, and dithionite was added in a volume of 20 µl of a 1 M phosphate buffer at pH 7.4 solution, saturated with dithionite. The SMP buffer is described in the Materials and Methods section.

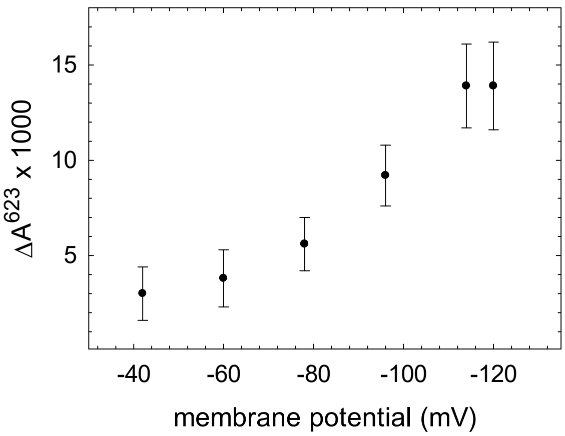

Supplementary Figure 2. Calibration of the oxonol VI absorbance signal. Asolectin liposomes of the same size as the SMPs were prepared with 0.5 mM KCl on the inside. The concentration of KCl on the outside was gradually increased: 2.5, 5, 10, 20, 40 and 50 mM and the absorbance change at 623 nm was measured after each addition. The standard deviation is calculated from eight measurements. Experimental conditions: 20 mM Hepes pH 8, 150 mM NaCl, 0.5 mM KCl, 2 µM oxonol VI and 1 µM valinomycin. The lipid concentration was 8 mg/ml and final volume was 1 ml.
